# Gender disparities in diabetes and coronary heart disease medication among patients with type 2 diabetes: results from the DIANA study

**DOI:** 10.1186/1475-2840-11-88

**Published:** 2012-07-27

**Authors:** Heike U Krämer, Elke Raum, Gernot Rüter, Ben Schöttker, Dietrich Rothenbacher, Thomas Rosemann, Joachim Szecsenyi, Hermann Brenner

**Affiliations:** 1 Division of Clinical Epidemiology and Aging Research, German Cancer Research Center, Im Neuenheimer Feld 581, D-69120, Heidelberg, Germany; 2 Practice of General Medicine, Blumenstrasse 11, D-71726, Benningen/ Neckar, Germany; 3 Institute of Epidemiology and Medical Biometry, Ulm University, Helmholtzstr. 22, D-89081, Ulm, Germany; 4 Department of General Practice and Health Services Research, University of Zürich, University Hospital of Zürich, Pestalozzistrasse 24, CH-8091, Zürich, Switzerland; 5 Department of General Practice and Health Services Research, University Hospital Heidelberg, Vossstrasse 2, Geb. 37, D-69115, Heidelberg, Germany

**Keywords:** Medical management, Diabetes mellitus, Cardiology

## Abstract

**Background:**

Coronary heart disease (CHD) is one of the most common long-term complications in people with type 2 diabetes. We analyzed whether or not gender differences exist in diabetes and CHD medication among people with type 2 diabetes.

**Methods:**

The study was based on data from the baseline examination of the DIANA study, a prospective cohort study of 1,146 patients with type 2 diabetes conducted in South-West Germany. Information on diabetes and CHD medication was obtained from the physician questionnaires. Bivariate and multivariate analyses using logistic regression were employed in order to assess associations between gender and prescribed drug classes.

**Results:**

In total, 624 men and 522 women with type 2 diabetes with a mean age of 67.2 and 69.7 years, respectively, were included in this analysis. Compared to women, men had more angiopathic risk factors, including smoking, alcohol consumption and worse glycemic control, and had more often a diagnosed CHD. Bivariate analyses showed higher prescription of thiazolidinediones and oral combination drugs as well as of angiotensin-converting enzyme (ACE) inhibitors, calcium channel blockers and aspirin in men than in women. After full adjustment, differences between men and women remained significant only for ACE inhibitors (OR = 1.44; 95%-confidence interval (CI): 1.11 – 1.88) and calcium channel blockers (OR = 1.42, 95%-CI: 1.05 – 1.91).

**Conclusions:**

These findings contribute to current discussions on gender differences in diabetes care. Men with diabetes are significantly more likely to receive oral combination drugs, ACE inhibitors and calcium channel blockers in the presence of coronary heart disease, respectively. Our results suggest, that diabetic men might be more thoroughly treated compared to women. Further research is needed to focus on reasons for these differences mainly in treatment of cardiovascular diseases to improve quality of care.

## Background

Type 2 diabetes mellitus (T2DM) is a major public health concern. It induces macro- and microvascular damage promoting long-term complications, like coronary heart disease (CHD), stroke or diabetic nephropathy, and is associated with significant morbidity and mortality [1,2]. The risk of CHD and stroke is altered by age, gender, insulin and glycemic control in patients with diabetes mellitus [3], but gender-specific differences in the prevalence of cardiovascular diseases (CVD) might also decrease with rising age, especially in older women with diabetes compared to men of the same age [4].

Diabetes and CVD treatment is complex: besides the different applicable agents, disease status, comorbidities, self-management capabilities and individual compliance of patients have to be considered by the treating physicians [5,6]. Diabetes treatment is generally intensified if CVD risk factors or comorbidities, such as hypertension, hypercholesterolemia or CHD, are present and vice versa [7,8].

However, there is evidence that women tend to receive a less adequate therapeutic management than men [9,10]. Until now, it is still unclear to what extent these gender differences can be explained by confounding factors, such as age, diabetes duration, adherence, prevalent depression or marital status [1,11].

We aimed to analyze whether or not gender disparities exist in diabetes and CHD medication after controlling for the most important confounding factors in an outpatient population of diabetic patients in Germany.

## Methods

### Study design and study population

This analysis is based on data from the baseline examination of the DIANA study (Type 2 Diabetes Mellitus: New Approaches to Optimize Medical Care in General Practice). DIANA is an epidemiological prospective cohort study with patients with T2DM conducted in the Ludwigsburg-Heilbronn area located in South-West Germany. The study was initiated in 2008 to address (short- and long-term) diabetes-related outcomes and to evaluate potentials for health care improvements in people with T2DM. People with a physician diagnosed T2DM aged 18 and older were recruited consecutively according to a standardized protocol by 38 general practitioners (GP) during regular practice visits between October 2008 and March 2010. The study protocol was approved by the Ethics Committees of the medical faculty of the University of Heidelberg and of the Chamber of Physicians of Baden-Württemberg.

### Data collection

Participating patients completed a self-administered standardized questionnaire at baseline. Information related to diabetes and other medical conditions was reported by the attending physician through a standardized questionnaire. GPs reported all diabetes-relevant physician-diagnosed comorbidities (‘yes’/ ‘no’) and submitted a complete list of all medications currently prescribed. Diabetes medication and CHD medication were classified according to the Anatomic Therapeutic Chemical (ATC) classification system (for more detailed information on classification see Additional file 1).

A blood sample was collected by the GP at time of recruitment and glycated haemoglobin A1c (HbA_1c_) was assessed by a central laboratory, using ion exchange high pressure liquid chromatography (HPLC) (G8, Tosoh Biosciences).

### Definition of key variables

For the following variables information from the GP questionnaire was used and they were defined accordingly: body mass index (BMI) in kg/m², most recent high density lipoprotein level (HDL) in mg/dl and blood pressure (systolic/ diastolic) in mmHg, duration of diabetes and participation in a disease management program for T2DM (DMP-DM). CHD was defined as prevalent CHD or past myocardial infarction. Antidiabetic drugs were differentiated in biguanide, sulfonylurea, alpha-glucosidase inhibitor, thiazolidinedione, glinide, glucagon-like peptide-I (GLP-I) analogue exenatide, dipeptidyl peptidase-4 (DPP-4) inhibitor, oral combination drug (counted as one drug) and insulin treatment in general. Insulins were further specified in short acting human insulin, intermediate acting insulin (basal insulin), (human) insulin combination (short and intermediate acting) and insulin analogue. CHD medication was differentiated in antihypertensive drug, i.e. angiotensin-converting enzyme (ACE) inhibitor, diuretic, beta-blocker, calcium channel blocker and other hypertensive drug, such as angiotensin II receptor blocker, lipid lowering medication and aspirin (see Additional file 1).

The following information was obtained form the participant questionnaires: age at time of recruitment, gender, level of school education, marital status, occupational status, smoking history and alcohol consumption as well as number of appointments with the GP. Information on participants’ self-estimated adherence to all prescribed medications was obtained by the 4-item self-report Morisky medication adherence questionnaire developed [12]. The sum score was calculated ranging from 0 (full adherence) to 4 (poor adherence). Patients were grouped as having a good (zero points), moderate (1 to 2 points) or poor adherence (3 to 4 points). The general health status was evaluated by the first question of the short-form-12 (SF-12) questionnaire [13].

Classification of glycemic control level was based on baseline HbA_1c_, defining ≤ 6.4% as good, 6.5% - 7.4% as moderate and ≥7.5% as poor [14].

### Statistical analysis

When information from the GPs’ was not available (only 3.8% of the participants), information from the participants’ questionnaires was used to minimize missing values, since we found very good agreement of both sources for participants for whom the information of both questionnaires was available (kappa coefficients for medications: >0.90 and for comorbidities: >0.80).

Descriptive statistics included 2-tailed t-tests for means and χ²-tests for proportions comparing differences between men and women. Analyzed covariates were socio-demographic characteristics, glycemic control, smoking status, alcohol consumption, diabetes duration, participation in a DMP-DM and comorbidities. Analyses on prescribed diabetes medication were stratified for gender and differentiated between the presence and absence CHD. Analyses on prescribed CHD medication were stratified for gender and restricted to participants with prevalent CHD. Logistic regression was employed to estimate unadjusted and adjusted odds ratios (ORs) and corresponding 95% confidence intervals (95%-CIs) for describing the association between gender and use of diabetes or CHD medication. In order to adjust for the main independent determinants, variables for multivariate logistic regression models were selected by backward selection separately for each medication group (diabetes and CHD medications). Variables with a p-value < 0.1 were kept in the model to limit potential confounding. Statistical testing was two-sided, an alpha level of 5% was applied, and SAS 9.2 (SAS Institute, Cary, N.C., USA) was used throughout.

## Results

Overall, 624 men (54.4%) and 522 women (45.6%) participated in this study (Table 1). On average, men were younger than women. Gender-specific differences also were found for other socio-demographic factors: women had a lower educational level, were more often singles and less often still employed. Smoking and alcohol consumption was far more prevalent in men than in women. Tentatively, more men than women showed a HbA_1c_ ≥ 7.5%. Men had significantly lower mean HDL levels than women. No gender differences were found for mean systolic or diastolic blood pressure. The number of GP appointments did not differ significantly between women and men. Self-reported medication adherence and self-rated general health status were similar between women and men. Men had significantly more often physician-diagnosed CVD and diabetes-related comorbidities, including CHD, intermittent claudication, stroke and diabetic nephropathy, whereas women had significantly more often a diagnosed depression.

**Table 1:** Description of the study population

	**Men**		**Women**		**p-value**
	**(n = 624)**		**(n = 522)**		
**Variables of interest**	**n**	**%**	**n**	**%**	
Age in years (mean, SD*)	67.2 (10.1)		69.7 (10.5)		
**Age in years**					
≤59	**137**	**22.0**	**84**	**16.1**	
60 - 69	**193**	**30.9**	**129**	**24.7**	
70 - 79	**239**	**38.3**	**231**	**44.3**	
≥80	**55**	**8.8**	**78**	**14.9**	**0.0001**
**Years of school education**					
≤9	**433**	**70.9**	**395**	**76.9**	
10 - 12	**104**	**17.0**	**94**	**18.2**	
≥13	**74**	**12.1**	**25**	**4.9**	**0.0001**
**Marital status**					
Single/ widowed/ divorced	**112**	**18.1**	**209**	**40.2**	
Married	**508**	**81.9**	**311**	**59.8**	**<0.0001**
**Occupational status**					
Employed	**158**	**26.5**	**78**	**15.7**	
Retired	**403**	**67.5**	**318**	**63.9**	
Housewife	**0**	**0**	**80**	**16.1**	
Other	**36**	**6.0**	**22**	**4.4**	**<0.0001**
**Smoking history**					
Never	**174**	**28.0**	**375**	**72.0**	
Ex-smoker	**355**	**57.2**	**100**	**19.2**	
Current smoker	**92**	**14.8**	**46**	**8.8**	**<0.0001**
**Alcohol consumption**					
Abstainer	**139**	**22.3**	**283**	**54.2**	**<0.0001**
**Body mass index (kg/m²)**					
<25	76	12.2	78	14.9	
25 - <30	255	40.9	187	35.8	
30 - <35	194	31.1	161	30.8	
≥35	98	15.8	96	18.4	0.19
**Glycemic control by HbA**_**1c**_					
Good (≤6.4%)	241	38.6	213	41.1	
Moderate (6.5 - 7.4%)	239	38.3	212	40.9	
Poor (≥7.5%)	144	23.1	93	18.0	0.10
**Mean HbA**_**1c**_**(SD) in **%	6.9 (1.1)		6.8 (1.0)		0.14
**Mean HDL (SD) in mg/dl**	47.7 (14.3)		57.3 (17.2)		**<0.0001**
**Mean systolic BP (SD) in mmHg**	137.8 (18.1)		136.8 (19.0)		0.41
**Mean diastolic BP (SD) in mmHg**	79.4 (10.2)		79.6 (10.5)		0.68
**Physician reported time since diabetes diagnosis**					
≤5 years	241	38.6	213	40.8	
6 – 10 years	185	29.7	146	28.0	
11 – 15 years	104	16.7	81	15.5	
≥16 years	94	15.1	82	15.7	0.82
**Appointments with GPs (last 3 months)**					
≤1	260	41.7	197	37.9	
2 – 3	245	39.3	216	41.5	
≥4	119	19.1	107	20.6	0.43
**Participation in a DMP-DM**	471	79.4	398	81.7	0.34
**Medication adherence**					
Good	455	75.6	385	77.0	
Moderate	132	21.9	102	20.4	
Poor	15	2.5	13	2.6	0.83
**General health status (self-rated)**					
Excellent or very good	62	10.0	40	7.7	
Good	366	58.7	291	55.8	
Fair	174	27.9	172	32.9	
Poor	21	3.4	19	3.6	0.21
**Physician diagnosed comorbidities**					
Hypertension	488	78.3	407	78.1	0.93
Hypercholesterolemia	350	56.2	300	57.8	0.58
Coronary heart disease	**147**	**23.6**	**67**	**12.8**	**<0.0001**
Heart failure	75	12.0	64	12.3	0.89
Intermittent claudication	**96**	**15.4**	**36**	**6.9**	**<0.0001**
Stroke	**47**	**7.5**	**22**	**4.2**	**0.02**
Nephropathy	**76**	**12.2**	**45**	**8.6**	**0.05**
Retinopathy	51	8.2	35	6.7	0.35
Neuropathy	144	23.1	104	20.0	0.20
Depression	**67**	**10.7**	**97**	**18.6**	**0.0002**
Cancer	63	10.1	49	9.4	0.70

*SD = standard deviation; HbA_1c_ = glycated haemoglobin A1c; GP = general practitioner;

DMP-DM = disease management program for treatment of patients with type 2 diabetes.

Missing values: education: 21, marital status: 6, occupational status: 51, smoking: 4, BMI: 1, HbA1c: 4, HDL: 270, systolic and diastolic BP: 70, appointments with GPs: 2, DMP-DM: 66, adherence: 44, health status: 1, hypertension: 2, hypercholesterolemia: 4, CHD: 2, heart failure: 1, intermittent claudication: 1, stroke: 2, nephropathy: 1, retinopathy: 1, depression: 1, cancer: 1

Significant results (p < 0.05) by χ² test are printed bold.

**Figure 1:**
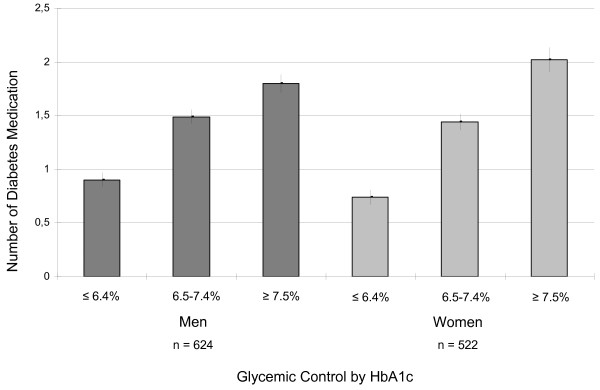
Mean number (± standard error) of diabetes medication by glycemic control.

**Figure 2:**
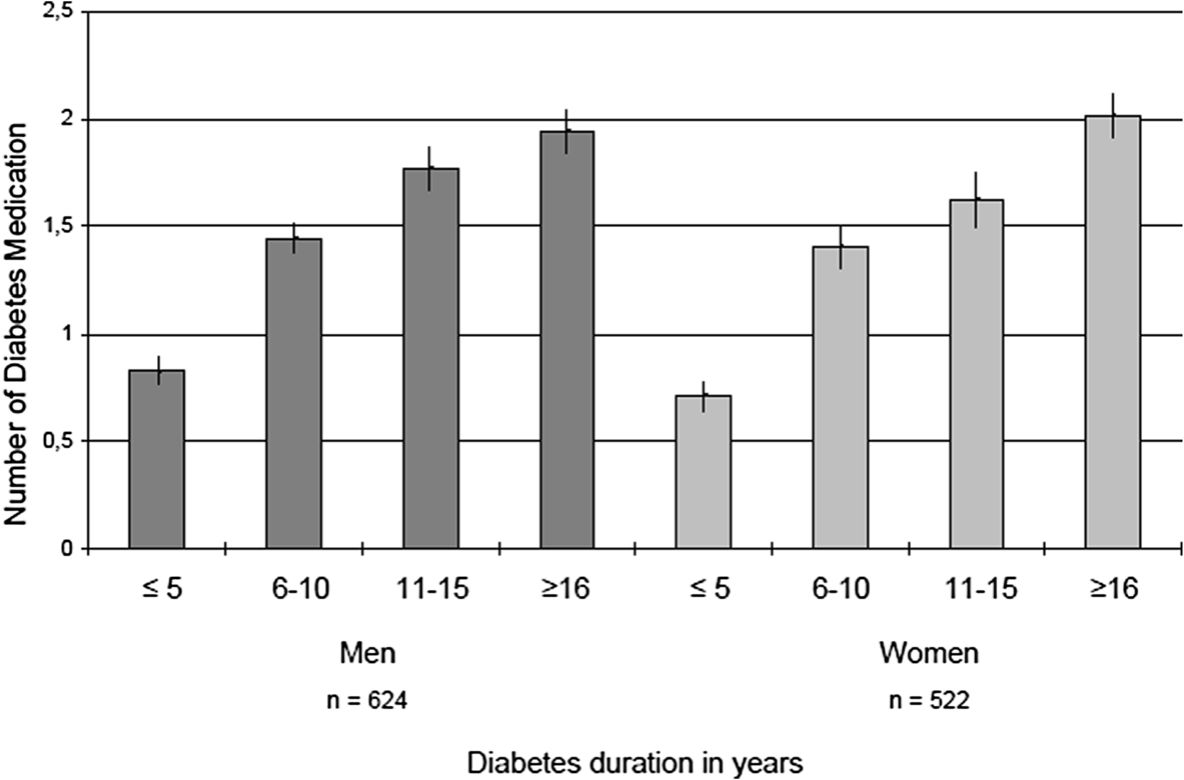
Mean number (± standard error) of diabetes medication by diabetes duration.

Characteristics of diabetes medication stratified for gender and prevalent coronary heart disease are described in Table 2. Significantly more men than women took at least one diabetes medication (men: 77.7% vs. women: 69.6%, p = 0.01). The most frequently prescribed diabetes drugs in men and women were biguanides, sulfonylureas and insulins. The prescription of more expensive diabetes drugs, such as GLP-I analogues, DPP-4 inhibitors and oral combination drugs, was rather low among our study participants. Statistically significant gender-specific differences were evident for thiazolidinediones (p = 0.04) and oral combination drugs (p = 0.02). Overall, prescription of insulin (insulin or insulin analogue) was almost equally frequent among men and women, but was far more common among patients with CHD than without. The number of diabetes medications was statistically significantly increasing with level of glycemic control in men and women (Figures 1 and 2).

**Table 2:** Diabetes medication by gender and prevalent coronary heart disease

**Medication of interest**					**With coronary heart disease**				**Without coronary heart disease**			
	**Men**		**Women**		**Men**		**Women**		**Men**		**Women**	
	**(n = 624)**		**(n = 522)**		**(n = 147)**		**(n = 67)**		**(n = 477)**		**(n = 455)**	
	**n**	**%**	**n**	**%**	**n**	**%**	**n**	**%**	**n**	**%**	**n**	**%**
Number of diabetes medication (mean, STD*)	1.3 (1.1)		1.3 (1.2)		1.5 (1.3)		1.4 (1.1)		1.3 (1.1)		1.2 (1.2)	
**Number of diabetes medication**												
0	**138**	**22.3**	**158**	**30.4**	27	18.5	15	22.7	**111**	**23.5**	**143**	**31.6**
1	**245**	**39.6**	**175**	**33.7**	50	34.3	22	33.3	**195**	**41.2**	**153**	**33.8**
2	**159**	**25.7**	**113**	**21.8**	46	31.5	17	25.8	**113**	**23.9**	**96**	**21.2**
3 and more	**77**	**12.4**	**73**	**14.1**	23	15.7	12	18.2	**54**	**11.4**	**61**	**13.5**
**Biguanide**	343	55.0	273	52.3	70	47.6	35	52.2	273	57.2	238	52.3
**Sulfonylurea**	132	21.2	92	17.6	32	21.8	14	20.9	100	21.0	78	17.1
**Alpha-glucosidase inhibitor**	14	2.2	13	2.5	5	3.4	1	1.5	9	1.9	12	2.6
**Thiazolidinedione**	**39**	**6.3**	**19**	**3.6**	11	7.5	3	4.5	28	5.9	16	3.5
**Glinide**	21	3.4	13	2.5	6	4.1	1	1.5	15	3.1	12	2.6
**Glucagon-like peptide-I analogue (GLP-I) exenatide**	6	1.0	5	1.0	4	2.7	0	0	2	0.4	5	1.1
**Dipeptidyl peptidase-4 (DPP-4) inhibitor**	20	3.2	28	5.4	7	3.3	4	6.0	17	3.6	24	5.3
**Oral combination drug**	**26**	**4.2**	**9**	**1.7**	6	4.1	1	1.5	**20**	**4.2**	**8**	**1.8**
**Insulin treatment**	123	19.8	107	20.5	43	29.7	20	29.8	80	16.8	87	19.1
**Short acting insulin**	47	7.5	40	7.7	16	10.9	7	10.4	31	6.5	33	7.3
**Intermediate acting (basal insulin)**	53	8.5	56	10.7	19	12.9	10	14.9	34	7.1	46	10.1
**Human insulin combination (short and intermediate acting)**	22	3.5	14	2.7	8	5.4	4	6.0	14	2.9	10	2.2
**Insulin analogue**	58	9.3	43	8.2	22	15.0	8	11.9	36	7.5	35	7.7

*SD = standard deviation. Number may not always add up to total because of missing values for some items.

Significant results (p < 0.05) by χ²-test are printed bold.

Table 3 shows the prevalence of CHD medication stratified by gender. Statistically significant gender-specific differences were found for ACE inhibitors (p = 0.003), calcium channel blockers (p = 0.01) and aspirin (p = 0.002). In the group of CHD patients, statistically significant gender-specific differences were only evident for ACE inhibitors (p = 0.02) and calcium channel blockers (p = 0.004).

**Table 3:** Coronary heart disease medication by gender and prevalent coronary heart disease

**Medication of interest**	**All**				**With coronary heart disease**			
	**Men**		**Women**		**Men**		**Women**	
	**(n = 624)**		**(n = 522)**		**(n = 147)**		**(n = 67)**	
	**n**	**%**	**n**	**%**	**n**	**%**	**n**	**%**
**Antihypertensive drug**	507	81.3	412	78.9	144	98.0	63	94.0
** Angiotensin-converting enzyme (ACE) inhibitor**	**431**	**69.1**	**316**	**60.5**	**120**	**81.6**	**45**	**67.2**
** Diuretic**	186	29.9	176	33.7	70	47.9	34	50.7
** Beta-blocker**	267	42.8	228	43.7	112	76.2	48	71.6
** Calcium channel blocker**	**168**	**26.9**	**106**	**20.3**	**50**	**34.0**	**10**	**14.9**
**Lipid lowering drug**	277	44.4	216	41.4	110	74.8	45	67.2
**Aspirin**	**213**	**34.1**	**133**	**25.5**	102	69.4	45	67.2

Number may not always add up to total because of missing values for some items. Significant results (p < 0.05) by χ²-test are printed bold.

The results of logistic regression analyses regarding the association between gender and diabetes and CHD medications with significant gender differences (p < 0.05) are described in Table 4. Men had 53% increased odds to receive any antidiabetic drug compared to women (95%-CI: 1.17 – 1.99, no table). Men compared to women had 1.8 and 2.5 higher odds to receive thiazolidinediones and oral combination drugs. After stratification for CHD, no significant gender-specific difference in diabetes medication was seen. After full model adjustment men had still 44% and 42% increased odds to receive ACE inhibitors and calcium channel blockers. A positive association between male gender and antithrombotic therapy with aspirin was also observed. In participants with CHD, gender-specific prescription differences of ACE inhibitors and calcium channel blockers were particularly strong. Significant gender differences in ACE inhibitors in CHD patients with diabetes sustained after adjusting for age, Hba_1c_, appointements with GP, medication adherence and comorbidities that are indications for renin-angiotensin system (RAS) blockade. Overall, all logistic regression models showed a very good model fit by Hosmer-Lemeshow test.

**Table 4:** Results of diabetes and coronary heart disease medication: Odds ratios (95% confidence intervals) for men compared to women (reference)

		**Total**	**With coronary heart disease**	**Without coronary heart disease**
**Diabetes Medication**				
**Thiazolidinedione**	**Crude**	1.77 (1.01 - 3.09)	1.73 (0.47 - 1.58)	1.71 (0.91 - 3.21)
	**Adjusted**^**a**^	1.75 (0.99 - 3.08)	1.58 (0.41 - 6.07)	1.68 (0.89 - 3.17)
	**Adjusted**^**b**^	1.42 (0.79 - 2.57)^1^	0.94 (0.28 - 3.17)^2^	1.52 (0.81 - 2.85)^3^
**Oral combination drug**	**Crude**	2.48 (1.51 - 5.34)	2.81 (0.33 - 23.79)	2.45 (1.07 - 5.61)
	**Adjusted**^**a**^	1.97 (0.94 - 4.13)	1.82 (0.21 - 15.96)	1.91 (0.82 - 4.45)
	**Adjusted**^**b**^	2.06 (0.98 - 4.31)^4^	1.93 (0.38 - 9.78)^5^	1.87 (0.84 - 4.13)^6^
**Coronary Heart Disease Medication**				
**Angiotensin-converting enzyme inhibitor**	**Crude**	1.46 (1.14 – 1.86)	2.17 (1.12 – 4.20)	
	**Adjusted**^**a**^	1.52 (1.18 – 1.95)	2.18 (1.09 – 4.35)	
	**Adjusted**^**c**^	1.44 (1.11 – 1.88)^7^	-	
	**Adjusted**^**d**^	-	2.61 (1.26 – 5.42)^8^	
**Calcium channel blocker**	**Crude**	1.45 (1.10 – 1.91)	2.94 (1.38 – 6.24)	
	**Adjusted**^**a**^	1.55 (1.17 – 2.06)	3.53 (1.58 – 7.89)	
	**Adjusted**^**c**^	1.42 (1.05 – 1.91)^9^	-	
	**Adjusted**^**d**^		3.64 (1.55 – 8.52)^10^	
**Aspirin**	**Crude**	1.52 (1.17 – 1.96)	1.10 (0.58 – 2.08)	
	**Adjusted**^**a**^	1.80 (1.38 – 2.36)	0.96 (0.49 – 1.87)	
	**Adjusted**^**e**^	1.36 (1.01 – 1.84)^11^	-	
	**Adjusted**^**f**^	-	1.12 (0.57 – 2.21)^12^	

Adjusted for: ^a^age and HbA_1c_ level, ^b^age, HbA_1c_ level, physician reported diabetes duration, appointments with the GP, marital status, diabetic nephropathy, depression, ^c^age, HbA_1c_ level, appointments with the GP, medication adherence, coronary heart disease, stroke, intermittent claudication, diabetic nephropathy and depression, ^d^age, HbA_1c_ level, appointments with the GP, medication adherence, stroke, intermittent claudication, diabetic nephropathy and depression, ^e^age, HbA_1c_ level, appointments with the GP, medication adherence, coronary heart disease, stroke, diabetic nephropathy and depression, ^f^age, HbA_1c_ level, appointments with the GP, medication adherence, stroke, diabetic nephropathy and depression.

Goodness of fit: ^1^p = 0.74; ^2^p = 0.87; ^3^p = 0.33; ^4^p = 0.66; ^5^p = 0.85 ^6^p = 0.66; ^7^p = 0.83; ^8^p = 0.46; ^9^p = 0.93; ^10^p = 0.70; ^11^p = 0.13; ^12^p = 0.76.

## Discussion

Our results demonstrate that men with diabetes were significantly more likely to receive oral combination drugs, ACE inhibitors and calcium channel blockers for CHD than women. Gender-specific differences could also be observed for aspirin use for the overall study sample, but not for the CHD subsample. Taken together, these patterns suggest that diabetic men might be more thoroughly treated than women, especially in terms of CHD medication. Furthermore, the number of diabetes medication was significantly increasing with the level of glycemic control and diabetes duration in men and women with diabetes.

Our finding of gender-specific, also not statistically significant differences in the prescription of diabetes medication such as thiazolidinediones is in accordance with the results of a German cross-sectional study [15]: Lehnert et al. analyzed data on 6,786 people with diabetes in primary care and found a slightly higher prescription rate of thiazolidinediones in men (4.4%) than in women (3.6%) above the age of 60 years. Apart from the different time frames of the studies (Lehnert et al.: 2001; our study: 2008–2010), differences in prescription rates might be mainly explained by disparities in diabetes duration (Lehnert et al.: 4.5 (±1.2 years) vs. our study: 8.8 (±7.1 years)) which is one of the main determinants of treatment choice. Reasons for the higher prescription of thiazolidinediones in men than in women are unclear. Studies have suggested that thiazolidinediones have beneficial effects on inflammatory and atherogenic parameters, blood pressure and microalbuminuria [16] and that thiazolidinediones doubles the risk of fractures among women, but not among men with diabetes [17]. Due to severe side effects the most frequently prescribed thiazolidinediones, namely Rosiglitazone and Pioglitazone, can no longer be prescribed for diabetes treatment in Germany since April 2011 [18], and Rosiglitazone was recommended for suspension by the European Medicines Agency (EMA) in September 2010 [19].

The higher prescription rate of oral combination drugs in men compared to women with T2DM might be explained by the higher number of comorbidities and burden of disease in participating men compared to women and the attempt of the general practitioners to reduce the overall number of medication especially for men.

Our findings regarding CHD medication are in line with previous research: The results of the Euro Heart Survey on Heart Failure including 8,914 patients with heart failure showed that women got less ACE inhibitors and beta-blockers than men [20]. A German cross-sectional study on 1,857 consecutive patients with heart failure likewise showed a difference in the prescription of ACE inhibitors and dosage of beta-blockers, and revealed a less evidence-based treatment in women than in men [21]. For patients with diabetes mellitus, a Swedish study including 229 primary care centres revealed - similar to our study - that men were more often prescribed ACE inhibitors than women and that only one third of the patients below 75 years was on lipid-lowering drug therapy [22]. The higher prescription rate of ACE inhibitors among men can potentially be explained not only by the higher CVD comorbidity, but also by the higher prevalence of nephropathy in men than in women and the protective effect of ACE inhibitors towards progression of nephropathy [23]. Brannström et al [24] investigated gender disparities in the pharmacological treatment of cardiovascular disease and diabetes mellitus in a cohort of elderly people aged 85 years or older. Women received significantly more drugs, more diuretics, more nitrates. In contrast to our study, no differences were found for insulin therapy, Aspirin and ACE inhibitors, but ACE inhibitor prescription rates were considerably lower in this study compared to ours. For patients with coronary events or revascularisation the EUROASPIRE III study found higher ACE-inhibitors prescription rates for men than for women. Women receive more diuretics, insulin and oral antidiabetic agents [25]. Lipid lowering treatment is generally recommended for all people with T2DM with a high risk for macroangiopathic complications or with CHD [26]. The underuse of lipid lowering drugs together with a lack of antithrombotic drugs might point to a suboptimal quality of health care, because both are crucial for the reduction of cardiovascular comorbidity and all-cause mortality in people with diabetes [27-29]. A large Danish primary care study [30] on patients screened for diabetes with a high risk for CVD and a large US retrospective study [31] on people with diabetes found that less women than men received an adequate lipid-lowering treatment. In our study which was conducted between 2008 and 2010, already 43% of the patients with diabetes received lipid lowering drugs, but with no differences between men and women.

In summary, as various studies [32,33] and a recently published review [34] point out, gender differences regarding diabetes mellitus are present for all steps in the glucometabolic pathway starting with differences in patho-physiological disturbances, the risk factor distribution, incidence and prevalence of complications, and finally diagnostic techniques and therapy. Franconi et al conclude, that a gender specific approach for all aspects of patient care is much needed to improve overall perspective of patients with diabetes mellitus [34].

Our study results are limited by the cross-sectional study design, i.e. causality cannot be derived and no conclusion on trends in treatment can be drawn. In particular, our study did not allow identifying with certainty the potential reasons underlying gender differences in medication. The relatively small numbers in each medication group limited the power to detect differences of small and moderate size. Although we aimed for the inclusion of all people with T2DM in a large number of practices, selection effects of participating GPs and regional variation in treatment might limit generalizability. Furthermore, the insurance status was not recorded at baseline. We could not perform a detailed monitoring of completeness of recruitment of diabetes patients in the practices.

## Conclusions

Men with diabetes were significantly more likely to receive oral combination drugs, ACE inhibitors and calcium channel blockers in the presence of coronary heart disease. These patterns suggest that diabetic men might be more thoroughly treated than women, especially in terms of CHD medication. We hope that our results will contribute to and stimulate the discussion on gender-specific disparities in diabetes healthcare.

## Competing interests

The authors declare that they have no competing interests.

## Authors’ contributions

HUK conducted the statistical analysis and HUK and ER drafted the manuscript. ER, GR, DR, TR, JS and HB designed the study, supervised its implementation and field activities. All contributing authors were revising the manuscript critically for important intellectual content. All authors have read and approved the final version of the manuscript.

## Supplementary Material

Additional file 1 Anatomic Therapeutic Classification (ATC) code. Click here for file

## References

[B1] Rivellese AA Riccardi G Vaccaro O Cardiovascular risk in women with diabetes Nutr Metab Cardiovasc Dis 2010 20 474 480 10.1016/j.numecd.2010.01.008 20621459

[B2] Lopez AD Mathers CD Ezzati M Jamison DT Murray CJ Global and regional burden of disease and risk factors, 2001: systematic analysis of population health data Lancet 2006 367 1747 1757 10.1016/S0140-6736(06)68770-9 16731270

[B3] Hayashi T Kawashima S Nomura H Itoh H Watanabe H Ohrui T Yokote K Sone H Hattori Y Yoshizumi M Ina K Kubota K the Japan Cholesterol and Diabetes Mellitus Investigation Group Age, gender, insulin and blood glucose control status alter the risk of ischemic heart disease and stroke among elderly diabetic patients Cardiovasc Diabetol 2011 10 86 10.1186/1475-2840-10-86 21978180 PMC3200162

[B4] Mercuro G Deidda M Piras A Dessalvi CC Maffei S Rosano GM Gender determinants of cardiovascular risk factors and diseases J Cardiovasc Med 2010 11 207 220 10.2459/JCM.0b013e32833178ed 19829128

[B5] Heisler M Piette JD Spencer M Kieffer E Vijan S The relationship between knowledge of recent HbA(1c) values and diabetes care understanding and self-management Diabetes Care 2005 28 816 822 10.2337/diacare.28.4.816 15793179

[B6] King DK Glasgow RE Toobert DJ Strycker LA Estabrooks PA Osuna D Faber AJ Self-efficacy, problem solving, and social-environmental support are associated with diabetes self-management behaviors Diabetes Care 2010 33 751 753 10.2337/dc09-1746 20150299 PMC2845021

[B7] American Diabetes Association (ADA) Standards of medical care in diabetes - 2010 Diabetes Care 2010 33 Suppl 1 11 61

[B8] Tschöpe C Schultheiss HP Diabetic cardiopathy: pathogenesis, diagnosis and therapy Internist 2003 44 806 818 [Article in German] 10.1007/s00108-003-0947-z 14631577

[B9] Ferrara A Mangione CM Kim C Marrero DG Curb D Stevens M Selby JV Translating Research Into Action for Diabetes Study Group. Sex disparities in control and treatment of modifiable cardiovascular disease risk factors among patients with diabetes: Translating Research Into Action for Diabetes (TRIAD) Study Diabetes Care 2008 31 69 74 10.2337/dc08-1082 17934157

[B10] Gouni-Berthold I Berthold HK Mantzoros CS Bohm M Krone W Sex disparities in the treatment and control of cardiovascular risk factors in type 2 diabetes Diabetes Care 2008 31 1389 1391 10.2337/dc08-0194 18375411 PMC2453666

[B11] Roe CM McNamara AM Motheral BR Gender- and age-related prescription drug use patterns Ann Pharmacother 2002 36 30 39 11816254 10.1345/aph.1A113

[B12] Morisky DE Green LW Levine DM Concurrent and predictive validity of a self-reported measure of medication adherence Med Care 1986 24 67 74 10.1097/00005650-198601000-00007 3945130

[B13] Ware J Jr Kosinski M Keller SD A 12-Item Short-Form Health Survey: construction of scales and preliminary tests of reliability and validity Med Care 1996 34 220 233 10.1097/00005650-199603000-00003 8628042

[B14] American Diabetes Association (ADA) Executive summary: standards of medical care in diabetes - 2010 Diabetes Care 2010 33 Suppl 1 4 10 10.2337/dc10-S004 PMC2797389 20042774

[B15] Lehnert H Wittchen HU Pittrow D Bramlage P Kirch W Böhler S Höfler M Ritz E Prevalence and pharmacotherapy of diabetes mellitus in primary care Dtsch Med Wochenschr 2005 130 323 328 [Arctile in German] 10.1055/s-2005-863050 15712019

[B16] Schernthaner G Forst T Gulba D Haberbosch W Hanefeld M Linss G März W Mehnert H Rosak C Schnell O Seufert J Tschöpe D Erdmann E Challenge in diabetes therapy: effects of glitazones beyond blood glucose control Dtsch Med Wochenschr 2009 134 949 954 [Article in German] 10.1055/s-0029-1220255 19384816

[B17] Loke YK Singh S Furberg CD Long-term use of thiazolidinediones and fractures in type 2 diabetes: a meta-analysis CMAJ 2009 180 32 39 10.1503/cmaj.080486 19073651 PMC2612065

[B18] Gemeinsamer Bundesausschuss (GBA) Arzneimittel-Richtlinie/ Anlage III (Glitazone zur Behandlung des Diabetes mellitus Type 2) http://www.g-ba.de/informationen/beschluesse/1141/. 02 July 2012

[B19] European Medicines Agency European Medicines Agency recommends suspension of Avandia, Avandamet and Avaglin http://www.ema.europa.eu/ema/index.jsp?curl=pages/medicines/general/general_content_000420.jsp&mid=WC0b01ac058001d126. 02 July 2012

[B20] Lenzen MJ Rosengren A op Reimer WJ S Follath F Boersma E Simoons ML Cleland JG Komajda M Management of patients with heart failure in clinical practice: differences between men and women Heart 2008 94 e10 10.1136/hrt.2006.099523 17575332

[B21] Baumhäkel M Müller U Böhm M Influence of gender of physicians and patients on guideline-recommended treatment of chronic heart failure in a cross-sectional study Eur J Heart Fail 2009 11 299 303 10.1093/eurjhf/hfn041 19158153 PMC2645055

[B22] Nilsson PM Theobald H Journath G Fritz T Gender differences in risk factor control and treatment profile in diabetes: a study in 229 swedish primary health care centres Scand J Prim Health Care 2004 22 27 31 10.1080/02813430310003264 15119517

[B23] Hasslacher C Wolf G Kempe P Ritz E Diabetic Nephropathie Diabetologie und Stoffwechsel 2010 5 Suppl 2 S113 S116 [Article in German]

[B24] Brannström J Hamberg K Molander L Lövheim H Gustafson Y Gender disparities in the pharmocological treatment of cardiovascular disease and diabetes mellitus in the very old Drugs Aging 2011 28 993 1005 10.2165/11594730-000000000-00000 22117097

[B25] Dallongville J De Bacquer D Heidrich J De Backer G Prugger C Kotseva K Montaye M Amouyel P Gender differences in the implementation of cardiovascular prevention measures after an acute coronary event Heart 2010 96 1744 1749 10.1136/hrt.2010.196170 20956490

[B26] Gaede P Lund-Andersen H Parving HH Pedersen O Effect of a multifactorial intervention on mortality in type 2 diabetes N Engl J Med 2008 358 580 591 10.1056/NEJMoa0706245 18256393

[B27] Kearney PM Blackwell L Collins R Keech A Simes J Peto R Armitage J Baigent C Cholesterol Treatment Trialists' (CTT) Collaborators Efficacy of cholesterol-lowering therapy in 18,686 people with diabetes in 14 randomised trials of statins: a meta-analysis Lancet 2008 371 117 125 10.1016/S0140-6736(08)60104-X 18191683

[B28] Wiggin TD Sullivan KA Pop-Busui R Amato A Sima AA Feldman EL Elevated triglycerides correlate with progression of diabetic neuropathy Diabetes 2009 58 1634 1640 10.2337/db08-1771 19411614 PMC2699859

[B29] Squizzato A Keller T Romualdi E Middeldorp S Clopidogrel plus aspirin versus aspirin alone for preventing cardiovascular disease Cochrane Database Syst Rev 2011 CD005158 21249668 10.1002/14651858.CD005158.pub3

[B30] Graversen L Christensen B Borch-Johnsen K Lauritzen T Sandbaek A General practitioners' adherence to guidelines on management of dyslipidaemia: ADDITION-Denmark Scand J Prim Health Care 2010 28 47 54 10.3109/02813430903335216 19929180 PMC3440615

[B31] Fu AZ Zhang Q Davies MJ Pentakota SR Radican L Seck T Underutilization of statins in patients with type 2 diabetes in US clinical practice: a retrospective cohort study Curr Med Res Opin 2011 27 1035 1040 10.1185/03007995.2011.567257 21410303

[B32] Leosdottir M Willenheimer R Persson M Nilsson PM The association between glucometabolic disturbances, traditional cardiovascular risk factors and self-rated health by age and gender: a cross-sectional analysis within the Malmö preventive project Cardiovasc Diabetol 2011 10 118 10.1186/1475-2840-10-118 22204568 PMC3270001

[B33] Moebus S Balijepalli C Lösch C Göres L von Stritzky B Bramlage P Wasem J Jöckel KH Age- and sex-specific prevalence and ten-year risk for cardiovascular disease of all 16 risk factors combinations of the metabolic syndrome –a cross-sectiona study Cardiovasc Diabetol 2010 9 34 10.1186/1475-2840-9-34 20696055 PMC2929217

[B34] Franconi F Campesi I Occhioni S Tonolo G Sex-gender differences in diabetes vascular complications and treatment Endocr Metab Immune Disord Drug Targets 2012 12 179 196 10.2174/187153012800493512 22236023

