# Supplementary material for: Gender disparities in diabetes and coronary heart disease medication among patients with type 2 diabetes: results from the DIANA study
Source: Cardiovasc Diabetol. 2012 Jul 27;11:88. doi: 10.1186/1475-2840-11-88 (PMC3526520; doi:10.1186/1475-2840-11-88)
Supplement: Additional file 1 — Anatomic Therapeutic Classification (ATC) code. [file 1475-2840-11-88-S1.doc]

**Appendix:** Anatomic Therapeutic Classification (ATC) code

| **Drug group** | **Anatomic Therapeutic Classification (ATC) code** |
| --- | --- |
| **Diabetes medication** |  |
| Biguanide | A10BA |
| Sulfonylurea | A10BB |
| Alpha-glucosidase inhibitor | A10BF |
| Thiazolidinedione | A10BG |
| Glinide | A10BX02 |
| Glucagon-like peptide-I (GLP-I) analogue  exenatide | A10BX04 |
| Dipeptidyl peptidase-4 (DPP-4) inhibitor | A10BH |
| Oral combination drug  (i.e. combination of biguanide and thiazolidinediones, biguanide and dipeptidyl peptidase-4 (DPP-4) inhibitor or sulfonylurea and thiazolidinediones) | A10BD |
| Insulin treatment in general | A10AB, A10AC, A10AD, A10AE |
| Short human acting insulin | A10AB01 |
| Intermediate acting insulin (basal insulin) | A10AC |
| (Human) insulin combination (short and intermediate acting) | A10AD01 |
| Insulin analogue | A10AB04, A10AB05, A10AD04, A10AD05, A10AE |
| **Coronary heart disease medication*** |  |
| Antihypertensive drug | C02, C03, C07, C08, C09 |
| Angiotensin-converting enzyme (ACE) inhibitor | C09 |
| Diuretic | C03 |
| Beta-blocker | C07 |
| Calcium channel blocker | C08 |
| Lipid lowering therapy | C10 |
| Aspirin | single drug: B01AC06, N02BA01; combination drug: B01AC30 |

* fixed combinations are listed under their respective ATC Code only
